# Genome-Wide Linkage Scan Identifies Two Novel Genetic Loci for Coronary Artery Disease: In GeneQuest Families

**DOI:** 10.1371/journal.pone.0113935

**Published:** 2014-12-08

**Authors:** Hanxiang Gao, Lin Li, Shaoqi Rao, Gongqing Shen, Quansheng Xi, Shenghan Chen, Zheng Zhang, Kai Wang, Stephen G. Ellis, Qiuyun Chen, Eric J. Topol, Qing K. Wang

**Affiliations:** 1 Heart Center, the First Affiliated Hospital, Lanzhou University, Lanzhou, Gansu 730000, P. R. China; 2 Center for Cardiovascular Genetics, Department of Molecular Cardiology, Lerner Research Institute, Cleveland Clinic, Department of Molecular Medicine, Cleveland Clinic Lerner College of Medicine of Case Western Reserve University, 9500 Euclid Ave., Cleveland, Ohio, 44195, United States of America; 3 Institute of Medical Systems Biology and School of Public Health, Guangdong Medical College, Dongguan, Guangdong, 523808, P. R. China; 4 Center for Cardiovascular Genetics, Department of Cardiovascular Medicine, Sydell and Arnold Miller Family Heart and Vascular Institute, Cleveland Clinic, 9500 Euclid Ave., Cleveland, Ohio, 44195, United States of America; 5 Scripps Translational Science Institute, Scripps Research Institute, Scripps Clinic, La Jolla, California, 92037, United States of America; 6 Center for Sleep Medicine, Neurological Institute, Cleveland Clinic, 9500 Euclid Ave., Cleveland, Ohio, United States of America; 7 Department of Genetics and Genome Sciences, Case Western Reserve University, 9500 Euclid Ave., Cleveland, Ohio, 44195, United States of America; 8 Key Laboratory of Molecular Biophysics of the Ministry of Education, College of Life Science and Technology and Center for Human Genome Research, Huazhong University of Science and Technology, Wuhan, P.R. China; University of Texas MD Anderson Cancer Center, United States of America

## Abstract

Coronary artery disease (CAD) is the leading cause of death worldwide. Recent genome-wide association studies (GWAS) identified >50 common variants associated with CAD or its complication myocardial infarction (MI), but collectively they account for <20% of heritability, generating a phenomena of “missing heritability”. Rare variants with large effects may account for a large portion of missing heritability. Genome-wide linkage studies of large families and follow-up fine mapping and deep sequencing are particularly effective in identifying rare variants with large effects. Here we show results from a genome-wide linkage scan for CAD in multiplex GeneQuest families with early onset CAD and MI. Whole genome genotyping was carried out with 408 markers that span the human genome by every 10 cM and linkage analyses were performed using the affected relative pair analysis implemented in GENEHUNTER. Affected only nonparametric linkage (NPL) analysis identified two novel CAD loci with highly significant evidence of linkage on chromosome 3p25.1 (peak NPL  = 5.49) and 3q29 (NPL  = 6.84). We also identified four loci with suggestive linkage on 9q22.33, 9q34.11, 17p12, and 21q22.3 (NPL  = 3.18–4.07). These results identify novel loci for CAD and provide a framework for fine mapping and deep sequencing to identify new susceptibility genes and novel variants associated with risk of CAD.

## Introduction

Coronary artery disease (CAD) is the leading cause of death globally, and accounts for >13% of all deaths (www.who.int) [1]. The number of people who die from heart disease is expected to increase to 9.8 million by 2030 (www.who.int). Long-term prospective clinical and epidemiological studies have identified several major risk factors for development of CAD, including smoking history, older age, male gender, high fat diet, personal history of angina pectoris, family history of myocardial infarction (MI), obesity, diabetes mellitus, high blood pressure, increased plasma total and low-density lipoprotein cholesterol, increased plasma triglycerides, and decreased plasma high-density lipoprotein cholesterol [2]–[4]. Genetic-epidemiologic studies suggest that family history is the most significant independent risk factor, which accounts for approximately 40% to 60% of the risk for CAD (i.e. an estimated heritability of 0.40–0.60) [5]–[12]. Identification of the genetic factors responsible for CAD might unravel novel biological pathways involved in development of CAD.

There are two major genome-wide, systematic, comprehensive and unbiased approaches to identify genes and genomic variants for a human disease, including genome-wide linkage studies (GWLS) using family samples and genome-wide association studies (GWAS) using population samples. There are advantages and disadvantages for both. GWAS are more powerful than GWLS to detect common alleles at a locus, but less powerful if the phenotypes of interest are due to the segregation of low-frequency or rare alleles [13]. Furthermore, whereas associations identified by GWAS can be due to spurious causes, especially heterogeneity/population stratification, family-based GWLS are not subject to such type 1 errors [13]. Recent GWAS by many large groups including our GeneID/Cardio-X team have identified about 50 loci for CAD [14]–[26]. However, it is estimated that the risk variants identified by GWAS may, in aggregate, capture only a small fraction (<20%) of the overall heritability of CAD [8], [27]. Thus, much of the heritability remains missing for CAD.

The major disadvantage of GWAS is that its discovering power is limited to common variants with relatively low risk (odds ratio or OR of 1.2 on average) [13]. In contrast, GWLS are potentially more powerful in detection of low frequency or rare variants, but with large effects on risk of the disease [13]. Therefore, it is highly likely that some of the remaining heritability for CAD may be associated with rare variants but with large effects. Because of the genetic heterogeneity of CAD/MI, GWLS in families could be much more powerful in identifying such rare variants. We consider a family study design to be more optimal for identification of rare genetic variants with large effects because the same mutation can be observed in many family members and the cause-effect relationship can be easily determined by co-segregation with the disease in the families. Genome-wide linkage scan is a traditional and classical approach to identify new regions of the genome that predispose to CAD. These genome-wide linkage scans with hundreds of sibling pairs have identified several major genetic susceptibility loci for CAD or MI, including loci on chromosome 1p34–36, 1q25, 2p13.1, 2q21.2–22, 2q36–q37.3, 3q13, 5q31, 7p14, 8p22, 13q12–13, 14q32.2, 15q26.2, 16q13, and Xq23–26 [3], [4], [28]–[30]. The loci identified by linkage analyses were much fewer than GWAS, suggesting that many more linkage loci remain to be identified with new CAD and MI families. Linkage analysis and GWAS have totally different mechanisms in detecting susceptibility loci, and it is highly possible that the linkage loci represent some of the missing heritability of CAD.

In this study, we report results from a genome-wide linkage scan from a well-characterized U.S. population with 428 nuclear families with early onset CAD and myocardial infarction (MI) (each family has at least two affected sibs and one unaffected sib, GeneQuest, mean pedigree size  = 5). Multiple new susceptibility genetic loci were identified for CAD, which provide new genetic bases of CAD and a framework to identify underlying susceptibility genes at each locus.

## Subjects and Methods

### GeneQuest Cohort of Families with Early-Onset CAD

The study subjects have been recruited in the United States through the Center for Cardiovascular Genetics and Department of Cardiovascular Medicine at the Cleveland Clinic [31]–[33]. Important demographic and epidemiological features are shown in Table 1 and as in previously published reports [31]–[33]. The present study has been approved by the Cleveland Clinic Institutional Review Board on Human Subject Research, and written informed consent was acquired from all participants.

**Table 1:** Clinical and Demographic Features of the Study Populations (GeneQuest)*.

Feature	GeneQuest
No. male/no. female	1152/878
Age at onset (mean ± SD)	44.4±9.7 years
No. affected with CAD/MI	974
Ethnicity:	
Caucasian	91.7%
African American	2.3%
Native American or Alaskan	1.7%
Hispanic	1.4%
Asian or Pacific Islander	0.8%
Mixed ethnicity	0.8%
Unknown ethnicity	1.3%
Pedigree structure:	
No. of pedigrees	428
Pedigree size (mean ± SD)	4.7±1.3
No. of relative pairs:	
Sibling/sibling	1,303
Sister/sister	258
Brother/brother	476
Brother/sister	569
Half sibling/half sibling	25

*From Wang et al and Shen et al [31], [33].

The GeneQuest study was the first large-scale prospective collection of families with early-onset CAD and MI for identifying the underlying genes. The GeneQuest cohort consists of 1,613 subjects from 428 multiplex families with early-onset CAD and MI. Each family has at least two affected sibs, and the majority of families also have one unaffected sibling. The details, including diagnostic criteria and ascertainment of the patients and their families have been described elsewhere [31]–[33]. In brief, the presence or absence of CAD was assessed according to a previous diagnosis of MI (based on the existence of at least two of the following: chest pain of ≥30 minutes duration, ECG patterns consistent with acute MI, or significant elevation of cardiac enzymes), history of revascularization procedures such as angioplasty or coronary artery bypass surgery (CABG), or patients undergoing treatment for angina pectoris. For patients in this cohort, the distribution of patients classified by the diagnostic criteria was 54.9% MI, 15.4% CABG, 14.9% angiography with >70% of stenosis, 12.2% PTCA (coronary artery bypass graft), and 2.6% other causes. The GeneQuest cohort had a total of 974 affected individuals recruited, and the average age at onset was 44.4±9.7 years. The epidemiological and demographic features were similar for both affected individuals and unaffected individuals. GeneQuest was characterized by predominantly males (n = 1,152) and white (91.7% for all the participants and 93.6% for those affected).

Patients with hypercholesterolemia, insulin-dependent diabetes, childhood hypertension, and congenital heart disease were excluded from this study.

### Isolation of Human Genomic DNA and Genotyping

Whole blood samples were drawn from each participant, and genomic DNA was isolated from the blood by use of the commercial Puregene Kits (Gentra).

Genotyping of microsatellite STR markers was performed by the National Heart, Lung, and Blood Institute (NHLBI) Mammalian Genotyping Service (directed by Dr. James L. Weber), Center for Medical Genetics at Marshfield Clinic by use of the Screening Set 11 consisting of 408 markers that span the entire human genome at every 10 cM [34]. DNA samples were genotyped for 1,163 well-characterized participants from 428 families of the GeneQuest cohort.

### Statistical Analysis

Before statistical analysis of genome-wide genotyping data, obvious pedigree errors, data errors, genotyping errors, and locus-order errors that commonly occur with a large-scale linkage analysis were corrected as described previously [13], [33], [35]. Allele frequencies for all markers used in the analysis were estimated in the GeneQuest population by a maximum likelihood method using the SAGE program FREQ [36]. We used RELTEST to analyze pedigree relationships and excluded 27 of 428 pedigrees with uncorrectable errors from further linkage analysis. RELTEST uses a Markov process model of allele sharing along the chromosome and classifies pairs of pedigree members according to their true relationship by use of genome-scan data [37]. We then used the S.A.G.E. program MARKERINFO to detect any Mendelian inheritance inconsistencies and excluded three families with inconsistent Mendelian inheritance from further analysis. Moreover, three pairs of monozygotic twins were identified and excluded from further statistical analysis. Only 388 Caucasian families were analyzed for linkage. To map new susceptibility loci for CAD, genome-wide nonparametric linkage (NPL) analysis was performed by use of the affected relative pair (ARP) analysis implemented in GENEHUNTER [38]. Like other ARP methods, the NPL statistic measures allele sharing among the affected individuals within a pedigree [38]. The scoring function statistic was used to evaluate, simultaneously, allele sharing among all those affected in a nuclear family, in contrast to pairwise comparison. Both two-point and multipoint NPL analyses were performed. This scoring function was asymptotically distributed as the Z statistic. The significance for linkage inference was determined by use of the criteria proposed previously [39], [40]. Specifically for the sib pair design, the NPL scores for suggestive, significant and highly significant linkage are 3.18 (corresponding to *P* value of 7.4×10^−4^ or LOD score of 2.2), 4.08 (*P* value of 2.2×10^−5^ or LOD score of 3.6) and 4.99 (*P* value of 3×10^−7^ or LOD score of 5.4), respectively.

## Results

**Figure 1:**
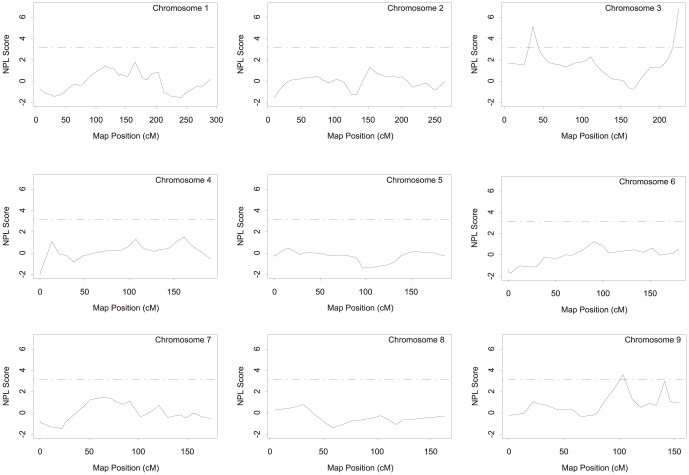
Genome-wide NPL scan for CAD-susceptibility loci on chromosomes 1–9 in the GeneQuest families. The vertical axis of each plot denotes NPL scores, and the horizontal axis represents map positions in cM from the telomere of the p arm to the telomere of the long arm of each chromosome. The horizontal solid line in each plot corresponds to an NPL score of 3.18 (P = 7.4×10^−4^), the criterion for suggestive linkage.

**Figure 2:**
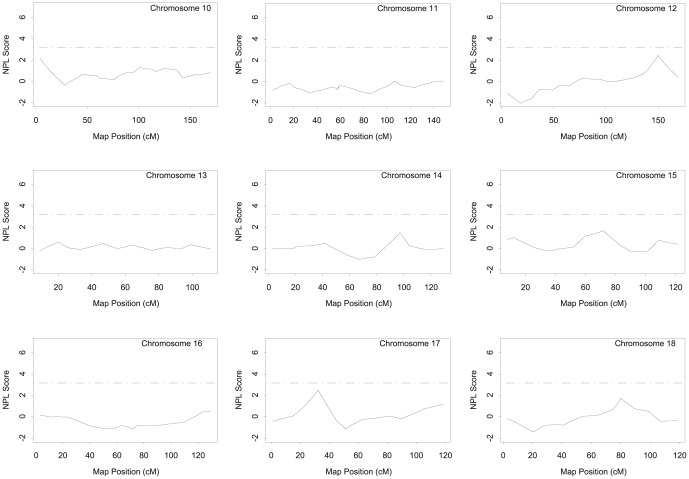
Genome-wide NPL scan for CAD-susceptibility loci on chromosomes 10–18 in the GeneQuest families.

**Figure 3:**
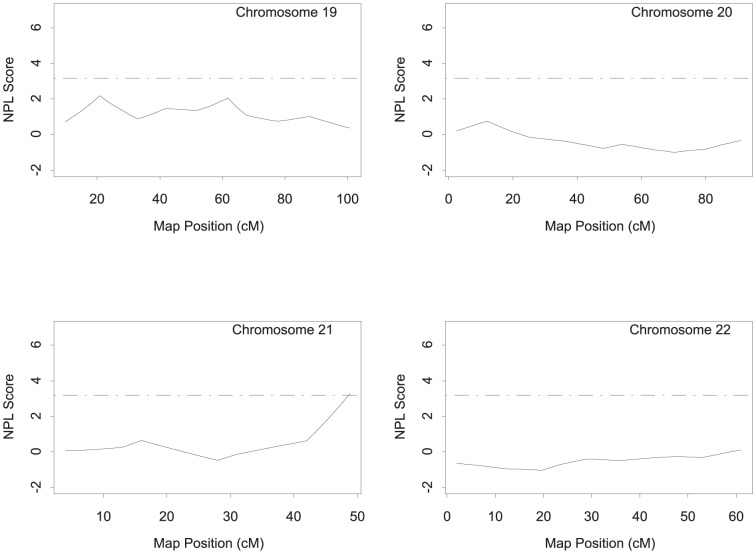
Genome-wide NPL scan for CAD-susceptibility loci on chromosomes 19–22 in the GeneQuest families.

Genome-wide NPL analysis of affected sibling pairs in the GeneQuest families was conducted using GENEHUNTER (http://www-genome.wi.mit.edu/ftp/distribution/software/genehunter/). The genomic regions with a peak two-point or multiple point NPL score that meets the suggestive linkage threshold (i.e. NPL ≥3.18) are listed in Table 2 and shown in Figs. 1–3. Six markers on four different chromosomes (chromosomes 3, 9, 17 and 21) produced an either two-point or multi-point NPL score of >3.18. The most significant linkage was identified for marker MFD427-AAA on chromosome 3q29 with a two-point NPL score of 6.54 and a multipoint NPL score of 6.84, which reached the genome-wide highly significant level (Figs. 1–3, Table 2). The second highly significant genome-wide linkage peak was also obtained on chromosome 3 (3p25.2), and had a two-point NPL score of 5.49 and a multipoint NPL score of 5.14 (Figs. 1–3, Table 2).

**Table 2:** Novel Genetic Loci for CAD Identified by GWLS in the GeneQuest Families.

Marker	Chromosomal Location	Map Position (cM)^a^	Physical Position (Mb)^b^	Two Point NPL	Multipoint NPL
D3S2403	3p25.1	37.2	13.17	**5.49**	**5.14**
MFD427-AAA	3q29	224.0	197.65	**6.54**	**6.84**
D9S910	9q22.33	104.0	101.62	3.72	3.61
AGAT125	9q34.11	141.8	131.95	3.69	2.99
ATA78D02	17p12	32.0	13.12	3.34	2.50
D21S1446	21q22.3	57.8	48.04	3.73	3.26

a Based on Marshfield Medical Genetics marker set 11.

b Based on either UCSC Genome Browser database or Marshfield Medical Genetics marker set 11.

Note that a marker with a two-point or multipoint NPL score of >4.99 (*P*<3×10^−7^) was considered to be linked to CAD highly significantly; A two-point or multipoint NPL score between 3.18 and 4.08 indicates suggestive linkage [38], [53].

The remaining four chromosomal regions on 9q22.33, 9q34.11, 17p12, and 21q22.3 generated evidence of suggestive linkage for CAD with either a two-point or multipoint NPL score of ≥3.18 (Table 2, Figs. 1–3).

## Discussion

In this study, we identified a list of new genetic loci linked to CAD using the whole-genome genotyping data from the GeneQuest nuclear families. The new CAD loci include two loci showing a high level of significance on the genome-wide level (3p25.2 and 3q29; NPL score of 4.99 or above) and four loci with suggestive linkage (9q22.33, 9q34.11, 17p12, and 21q22.3; NPL score of 3.18 to 4.07) (Tables 2, Figs. 1–3). These “potentially interesting” CAD linkages are novel. These results provide a framework for future fine mapping and deep sequencing for identifying new genomic variants and new susceptibility genes associated with risk of CAD. For example, future analysis of next generation sequencing data from CAD families may focus on the linkage regions first to identify genomic variants with potentially large effects on risk of familial CAD.

Using genotyping data from the same GeneQuest families, we previously identified a novel linkage for HDL-cholesterol, which spanned a 65 cM region, showed a bi-phasic pattern, and peaked at markers MFD433 and GATA131D09 on chromosome 3p25–26 [13]. The CAD locus represented by D3S2403 is on 3p25.2 and partially overlaps with the second, smaller peak of the HDL-C locus. Because a lower HDL-cholesterol level is a significant risk factor for CAD, it is possible that the same susceptibility gene may be responsible for HDL-cholesterol and CAD at the partially overlapping locus. The *PPARG* gene encoding peroxisome proliferator-activated receptor gamma is located within the CAD locus, and is a short distance of 697 kb from D3S2403. A variant in *PPARG* (rs1801282, P12A) was found to be associated with type 2 diabetes [41]–[43], which is a risk factor for CAD. A large-scale gene-centric meta-analysis showed that SNP rs12631819 in *PPARG* was associated with HDL-cholesterol levels [44]. The association between variants in *PPARG* and CAD was controversial because many studies reported inconsistent results. Meta-analysis showed that the H161T variant in *PPARG* was associated with risk of CAD under a fixed-effect model, but the association became non-significant under a random-effect model; the P12A variant was not associated with CAD [45]. Future studies are needed to assess whether *PPARG* is the gene responsible for CAD at the 3p25.2 locus. Moreover, the nearest gene from the CAD marker D3S2403 is *IQSEC1*, which encodes a protein with an IQ motif and a Sec domain. Interestingly, marker MFD427-AAA with the peak NPL score for the 3q29 CAD locus is located within the *IQCG* gene encoding another protein with an IQ motif. Therefore, *IQSEC1* and *IQCG* became candidate genes for CAD for the 3p25.2 and 3q29 loci for CAD, respectively.

The 3q29 locus for CAD was supported by several other studies showing positive but not significant linkage. For instance, meta-analysis of four genome-wide linkage studies for CAD indicated that the genetic region of 3q26-27 might contain susceptibility genes for CAD (*P* = 0.0001) [46]. Chromosome 3q27 was also suggested to be a CAD susceptible locus in Indo-Mauritians (LOD  = 2.13, p = 0.0009) [47]. The 3q29 CAD locus identified in this study may not be the same locus as the 3q26-27 locus because the two linkage peaks for the two loci are separated by 14 Mb and do not appear to overlap (Fig. 1 and Francke et al [47]). Chromosome 3q29 was found to be linked to atherogenic dyslipidemia [48]. Using the same genotyping data as in the present study, we previously identified a locus at 3q28-29 for HDL-cholesterol with LOD scores passed the threshold for suggestive significance [13]. Together, the tip of the long arm of chromosome 3 may harbor one or more novel susceptibility genes for CAD and its risk factors. In addition to the *IQCG* gene, the 3q29 CAD locus harbors several other possible candidate genes for CAD. The *LMLN* gene encodes an M8 family of metallopeptidase associated with lipid droplets and playing a role in lipid storage, generation of ROS, cell migration and invasion [49]. The *CEP19* gene encodes a 19 kDa centrosomal protein involved in obesity, glucose intolerance and insulin resistance [50]. The second interesting locus is the 9q22.33 CAD locus represented by marker D9S910. The finding of this locus was supported by careful comparison to linkage analysis in 42 families in a French Canadian population, which showed that our 9q22.33 CAD locus had a positive, but not significant, NPL score of 2.35 in the French Canadian population (Fig. 1 in [30]).

The linkages on chromosome 17 are of particular interest. A suggestive linkage for MI was previously reported on chromosome 17 at a position of 50.7 cM in a cohort with 739 affected sibpairs (LOD  = 2.85). The entire putative MI linkage spanned from marker D17S921 at 17q11.2 (40 cM) to D17S787 at 17q21 (79 cM) [28], and overlaps with the 17p12 locus identified in our study (Fig. 2).

We compared our linkage results to GWAS data reported to date. Interestingly, we found that SNP rs579459 in the *ABO* gene identified as a risk variant for CAD by GWAS is located 4.20 Mb from marker AGAT125 at the 9q34.11 region showing peak linkage to CAD in this study. It is important to note that in other CAD loci identified by GWLS in this study, GWAS did not identify any risk variants for CAD. There are several potential explanations. First, GWLS signals may represent variants or susceptibility genes for familial CAD, but not for sporadic CAD as used in GWAS. Second, GWLS signals may represent low frequency or rare variants with large effects on risk of CAD, which were generally ignored by GWAS. Considering the fact that larger meta-GWAS may not be easily practical for CAD now, re-focus on family-based studies may be the priority in the short future to identify new risk variants for CAD in our opinion.

The identification of new CAD linkage loci might have been facilitated by our strict criteria for pedigree recruitment, which focused on white families enriched with well-diagnosed early-onset CAD, and pre-empted common risk factors, such as hypercholesterolemia and insulin-dependent diabetes. Furthermore, compared with other reported genome-wide linkage scans for CAD and MI, our patient population has the youngest age at onset, <45 years in males and <50 years in females, which is expected to significantly increase the genetic component involved in CAD. Family history was another factor for us to recruit families, with aim to identify inheritable CAD cases and to exclude environmental ones. Seeking for families with strong and multi-generational CAD history remains the most accessible way of increasing the inherited component for the recruited familiar cohort [51]. Clinical studies suggest that individuals with a family history of CAD form a potential target groups for early aggressive primary prevention strategies [52].

Despite a great success in identifying new genetic loci for CAD in this study, several technical limitations associated with non-parametric linkage analysis should be well-recognized. First, the families were ascertained through probands with a strong family history of CAD, and by the unique selection strategy, which focused on white families enriched with well-diagnosed early-onset CAD. Such family recruitment may be subjected to some selection biases and some parameter estimates for CAD risk may not straightforwardly be extended to the general population. Therefore, a precaution is clearly needed for interpreting the risk estimate when translated into clinical practice. Second, due to very nature of non-parametric linkage analysis, our analysis did not model person-specific covariates to remove the effects of potentially confounding factors. Although some measures such as pre-empting common risk factors like hypercholesterolemia and insulin-dependent diabetes were taken during the family recruitment, some sporadic or environmental cases might be included in the analysis, causing identity-by-decent values between affected sib pairs downward-estimated, particular when gene-environment interactions are present. Third, due to limited sample sizes, we did not perform a stratified analysis by different subtypes of CAD and hence only genomic regions linked to general definition of CAD were identified. Fourth, this study is limited by inadequate marker density provided by microsatellite markers, fine mapping and analyses of genetic variants by use of high-density SNP markers or new-generation DNA sequencing technologies might be a better design in our future studies. Finally, there is discrepancy between our previous report of linkage to chromosome 1p34-36 [31]–[33] and novel linkages identified in this study, although both utilized the original 438 GeneQuest nuclear families. The discrepancy may be related to the different statistics involved in the SAGE program used in the previous report. The SAGE program in our previous study [33] analyzes all available information from the GeneQuest pedigrees, including both affected and unaffected family members, but the Genehunter program used in the present study analyzes only affected members in GeneQuest families. Furthermore, methodological differences might explain the discrepancies. In the previous report [33], we used a Haseman-Elston regression-based method implemented in the SAGE program, which models the squared phenotypic difference of a sib pair as function of genetic variance and linkage parameter. In the present study, the Genehunter program computes the averaged identical-by-decent sharing at a marker locus between affected sibs, and then the linkage inference is made by comparing this nonparametric statistic with its expectation. Therefore, it is not surprising that the statistical power for the two methods might be different under different scenarios. Moreover, the Haseman-Elston regression-based method can take into account of epidemiological covariates, as we did in the previous report [33], while the nonparametric method suffers from this limitation. Nevertheless, affected only analysis has a merit of more accurate phenotyping as these affected individuals are often subject to a strict diagnosis. In contrast, phenotyping for normal individuals for a late onset disease like CAD can be a difficult task as the presently unaffected individuals can become affected later in their life.

In conclusion, the present study has identified two novel genetic loci for CAD that passed the threshold of highly significant and four genetic loci with suggestive evidence of linkage. Future fine mapping and deep sequencing should facilitate the identification of an underlying major (or minor) gene for this complex disorder at each locus. Identification of the CAD gene(s) should uncover the biological pathways and molecular mechanisms for the pathogenesis of CAD.
